# Community Capacity Building for Physical Activity Promotion among Older Adults—A Literature Review

**DOI:** 10.3390/ijerph14091058

**Published:** 2017-09-13

**Authors:** Tobias Ubert, Sarah Forberger, Dirk Gansefort, Hajo Zeeb, Tilman Brand

**Affiliations:** 1 Gesundheitswirtschaft Nordwest e.V., 28195 Bremen, Germany; t.ubert@gwnw.de; 2 Leibniz Institute for Prevention Research and Epidemiology—BIPS, 28359 Bremen, Germany; forberger@leibniz-bips.de (S.F.); gansefort@leibniz-bips.de (D.G.); zeeb@leibniz-bips.de (H.Z.); 3 Research Focus Health Sciences Bremen, University of Bremen, 28359 Bremen, Germany

**Keywords:** physical activity, elderly, capacity building, community

## Abstract

Community-based interventions to promote physical activity (PA) among older adults are of high interest in health promotion since they promise to be effective strategies to reach this population group. Community capacity building, that is, the local promotion of knowledge, skills, commitment, structures, and leadership, is among the recommended core strategies. However, little guidance is provided on how to achieve a high degree of community capacity. This study aims to identify practical strategies to enhance community capacities for PA promotion among older adults (50 years or older) and to evaluate their success. A literature review was conducted using scientific databases (PsycInfo and Web of Sciences) and grey literature (national and international project databases), and 14 studies (16 articles) were identified. Five groups of capacity building strategies emerged from the literature: (1) building community coalitions and networks, (2) training of professionals, (3) training of laypersons, (4) strengthening competence and awareness in the target population, and (5) allocation of financial resources. All studies used more than one strategy. Coalition building and strengthening competence and awareness were most frequently used. Feasibility and acceptability of the capacity building strategies were demonstrated. However, intervention effects on PA behavior and other relevant outcomes were inconsistent. The one study that systematically compared different capacity building approaches did not find any evidence for beneficial effects of intensified capacity building. More rigorous research evaluating the efficacy of specific strategies to enhance community capacities for PA promotion is needed.

## 1. Introduction

Physical activity (PA) is an important factor for a healthy and independent life in older age [1,2,3,4,5]. Although the beneficial health effects of PA are widely known, levels of physical inactivity are high in high-income countries and increase with age [6]. For example, data from the German Health Interview and Examination Survey for Adults show that only 18% of the population aged 60–69 adhere to the WHO recommendations of at least 150 min of moderate to vigorous PA per week, and 36% of those in this age group are classified as physically inactive [7].

While behavior-oriented programs for PA promotion are prone to social selectiveness among the participants [8], community-based programs offer the potential for a more equitable and sustainable approach [9,10]. Communities (e.g., a geographical area or a municipality) are important settings for health promotion because people are reached in their natural living environment [11]. They can be understood as supersettings, with their capability to support and coordinate the health promotion activities of other settings within the community, such as schools or work places [12]. Moreover, a community-based approach can reach persons who are not integrated in these other settings, such as the unemployed or older adults. However, due to the large number of stakeholders involved and the complexity of the interventions, challenges in the use of community-based approaches arise [13].

The term capacity building has been applied in various fields and it is related to a number of concepts such as community development, empowerment, and community coalition building [14,15]. The WHO introduced community capacity building in the Jakarta Declaration in 1997 as one of the 21st century priorities for health promotion, which was an important step for establishing the concept as a core principle of health promotion worldwide [16]. It was subsequently added to the WHO health promotion glossary where it is defined as “the development of knowledge, skills, commitment, structures, systems, and leadership to enable effective health promotion” [17]. The definition explains that capacity building encompasses three levels of action: the advancement of knowledge and skills among practitioners, the expansion of support and infrastructure for health promotion in organizations, and the development of cohesiveness and partnerships for health in the community. Increasing community capacities is among the key strategies of community-based health promotion [14]. Community capacity building is sometimes regarded as an aim in itself, fostering the role of the community as the natural unit of solutions for diverse health needs [18,19]. Moreover, community capacities are also an important resource for implementing specific health promotion programs [20].

While scholars agree that capacity building is an important approach for health promotion, there is little guidance regarding the practical strategies to be adopted for capacity building. The aim of this review was to identify practical strategies for capacity building in the field of PA promotion for older adults. Furthermore, we wanted to find evidence of success of these strategies. Success referred to both implementation barriers and facilitators when applying the strategies, as well as their contribution to intervention outcomes.

## 2. Materials and Methods

A literature search was conducted in March 2017. Two scientific databases (PsycInfo, Web of Sciences) were searched using the following keywords: “capacity building”, “community development“, “community empowerment”, “community network*”, “coalition building”, “community capacity*”, “community building”, “community participation”, “community partnership*”, “community leadership”, “community coalition”, “increase”, “promote”, “enhance”, “improve”, “develop”, “build”, “community readiness”, “health promotion”, “prevention”, “elderly”, “older adults”, “senior”, “older people”, “ageing”, “communit*”, “municipalit*”, “environment*”, “neighborhood”, “third sector”. The keywords were combined using the Boolean operators OR and AND, as well as the truncation (“*”). A detailed description of the search strategy is provided in the supplementary material (Tables S1 and S2). Two national experts were asked to recommend journals for a manual search (Prof. Dr. Alf Trojan; Dr. Stefan Nickel).

Grey literature was searched in several national and international literature and project databases in the field of health promotion, including The Community Guide (USA), the University of York Centre for Reviews and Disseminations (UK), Kooperation für nachhaltige Präventionsforschung (cooperation for sustainable prevention research), Kooperationsverbund Gesundheitliche Chancengleichheit (cooperation network “Equity in Health”), IN FORM—Deutschlands Initiative für gesunde Ernährung und mehr Bewegung (Germany’s national initiative to promote healthy diets and physical activity) (all from Germany), quint-essenz (Switzerland), and Fonds Gesundes Österreich (Austrian Health Promotion Foundation) (Austria). Since there was no common structure within these databases, various strategies were used to identify publications using the above-mentioned keywords and Boolean operators.

Publications were included if (1) they were published between 1997 and February 2017; (2) written in German or English; (3) targeted older adults (50 years or older); (4) the intervention contained a PA component; and (5) the intervention contained capacity building activities as described in the WHO definition [17]. No restrictions were made with regard to the study design, comparison groups, or outcome parameters. Interventions that targeted a broader age range were not excluded as long as they also addressed older adults. Similarly, interventions with a clearly described PA component were also not excluded if outcomes other than levels of PA or physical fitness were assessed, such as fall incidence or weight loss, or if other intervention components were part of the program (e.g., nutrition, health education on other topics, health screenings).

The title and abstract of identified publications were screened by one reviewer and full texts of the included abstracts were reviewed independently by two reviewers. Disagreements were resolved through discussions with a third reviewer. Data extraction was conducted independently by two reviewers. In case of disagreements, a third reviewer was consulted. The following information was extracted from the included studies: author(s), study design, setting, target group, sample size, targeted health behavior, capacity building strategies, outcome measures, and results.

The critical appraisal form of the Stanford School of Medicine [21] was used for the quality assessment, except for project reports with no identifiable study design. The latter were treated as case studies and assessed using the critical appraisal tool of case studies developed by the Centers for Evidence-based Management [22]. The appraisal forms include 10 items, apart from the form for intervention trials (11 items). The quality assessment was performed individually by two reviewers. Positive ratings were summed up to indicate the overall quality.

The included studies were synthesized in a narrative fashion. Based on the descriptions in the included studies, we developed a set of categories to summarize the capacity building strategies. The categories distinguish between different kinds of activities and between different target groups of these activities (community networks, professionals, laypersons, and target population). The development of the categories was guided by the WHO definition of community capacity building cited afore.

## 3. Results

**Figure 1:**
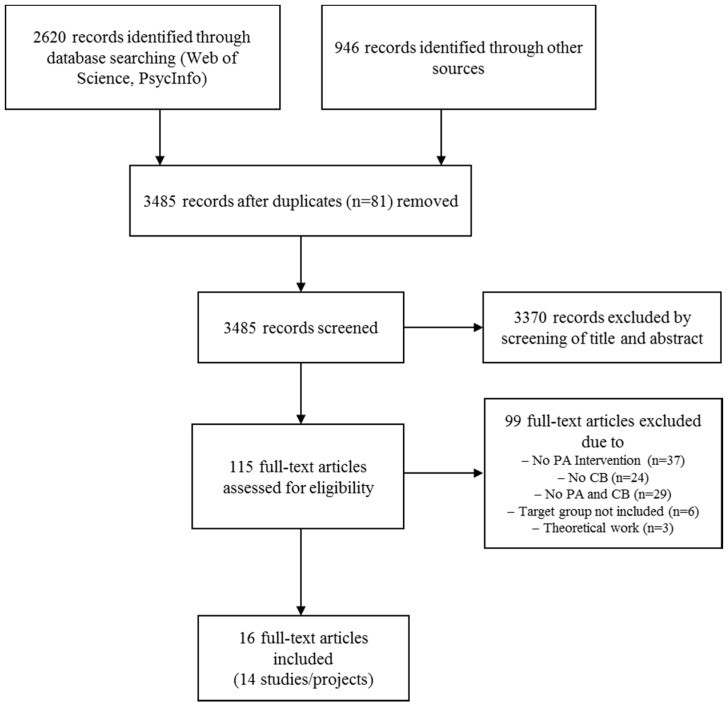
Flowchart of the literature search (PA physical activity; CB capacity building).

Of the 3485 initially identified records, 14 studies (16 publications) were included in the final analysis (Figure 1). The studies include five case studies, four cross-sectional studies (surveys), three cohort studies, and two cluster randomized controlled trials.

Most studies were of low to moderate quality, with four to six positive ratings out of 10 or 11 items (Table 1). Quality gaps appeared in items such as the reporting of an a priori hypothesis, the number of excluded persons or refusals before the study, or the sample size calculation for adequate statistical power. In particular, project reports often did not meet the standards of the respective assessment tools. They generally did not report methods employed for collecting data or whether quality control measures were used. The full results of the quality appraisal can be found in the supplementary material (Tables S3–S7).

Seven of the 14 studies were from the US, three from Austria, two from Germany, and one each from The Netherlands and Thailand. All studies were published between 2003 and 2016. In eleven studies, the settings were municipalities or geographic areas of varying size (communities, city districts, counties, “area of Vienna”), while in three, the setting was community organizations such as senior-citizen centers, community centers, or churches. Three studies made no specifications about the sample size. Ten studies set a specific age threshold as a target group criterion. Other target group criteria were ethnicity/migrant background, low socioeconomic status, limited mobility, high body mass index, or physical inactivity.

### 3.1. Capacity Building Strategies

We classified the capacity building strategies described in the included studies into five categories: (1) Community-based coalition and network building; (2) professional training in institutions and organizations; (3) training of laypersons; (4) strengthening competence and awareness in the target population; and (5) allocation of financial resources. All interventions used a combination of different capacity building strategies (Table 2).

Regarding the study objectives related to capacity building, two approaches could be distinguished: Some of the studies used capacity building to implement and disseminate an existing behavior change program such as the Diabetes Prevention Program [38], the Stepping On program [36], or the Arthritis Foundation Exercise Program [28]. In other studies, the development of local interventions was part of the capacity building process [23,24,25,26,35]. In several studies, the capacity building strategies were incorporated in a theoretical framework such as the Lewin’s concept of Rational Social Management [27], Intervention Mapping [33], Interactive Systems Framework [37], and Cooperative Planning Process [24].

#### 3.1.1. Community-Based Coalition and Network Building

Community-based coalition and network building was used in 12 studies. Coalitions and networks were often initiated by universities or federal organizations. Typically, a wide range of partners were involved, such as representatives from the municipalities, local health departments, ageing units, community organizations, ethnic minority groups, peers (older adults), and other stakeholders (e.g., sports clubs, tourism agency). The coalitions’ function and degree of formalization varied considerably. For example, Zgibor et al. described the development of a formalized partnership between a funding agency, a research institution, and local sites [28]. The scope of this partnership was restricted to the implementation of a behavior change program with clearly defined roles and responsibilities. In the study by Peterson et al., a research institution provided technical support, training, and consultation to local lead agencies to facilitate the implementation of a fall prevention program [37]. Other projects followed a more participatory approach where the community coalitions decided which preventative actions they would take [23,24,25,29,30,35]. In many cases, a core working group was established, complemented by a larger network of stakeholders. For example, in one project, a steering committee consisting of a university partner, the local senior office, and two community centers was formed from a larger round table network [30]. The steering committee developed the work plan and monitored the project progress. In some projects, these core working groups also decided upon the allocation of financial resources to small-scale local projects [25,35].

In two projects, a theoretical model structured the coalition building process [24,27]. For instance, the Building Policy Capacities for Health Promotion through Physical Activity among SEdentary Older People (PASEO) project used the four stages of the Cooperative Planning Process model (team building—alliance building—planning—implementation) to guide the process [15,24]. The coalition developed an action plan and prioritized activities. The action plan was then implemented at the local level. To ensure sustainability, the responsibility for the moderation of meetings and workshops was handed over step by step to the main coordinating partner “Wiener Gesundheitsförderung” (Health Promotion Vienna), a nonprofit organization. Likewise, researchers who initiated the coalition building process in other projects gradually withdrew their activities and handed over the responsibility to local partners [26,30,35].

#### 3.1.2. Training of Professionals

Training for professionals was provided in six projects and targeted either exercise class instructors or the management competencies of the local organizations. The training program was mostly delivered by scientists in a workshop format. For example, part of the Active Aging Community Task Force (AACTF) project was a training program for local instructors of exercise classes [32]. The content of the workshop was based on national curriculum standards for preparing older adult fitness instructors. As a result of these efforts, 416 local instructors attended the workshops and 153 new exercise classes for older adults were created.

Similarly, a strength training program was disseminated in local communities through train the trainer workshops (“leadership training”) involving 244 peers and professionals within the framework of the People Exercising Program [31]. The workshop curriculum included didactic sessions on exercise and ageing, as well as practical sessions on how to perform all the exercises and also correct common technique errors.

The project described by Sundermeier [26] was comprised of two training formats. Workshops on potentials for service optimization and public relations issues were offered to local service providers and administrative decision-makers. In addition, one-day training was offered to PA class instructors. This training focused on exercises for older inactive adults.

Reis-Klingspiegl [35] briefly described the “Cash & Coaching” format. Project staff from 13 participating communities could submit a proposal to get funding for local health promotion projects. When proposals were eligible for funding, applicants automatically received a coaching program in project management delivered by scientists.

#### 3.1.3. Training of Laypersons

Three studies used the training of laypersons as a practical strategy. The study by West et al. [38] investigated the efficacy of the Diabetes Prevention Program delivered by lay health educators (LHEs). LHEs received 32 h of face-to-face training on how to deliver a lifestyle intervention comprising key elements of a behavioral weight-control approach. Furthermore, they were trained in recruitment methods. The LHEs were community volunteers and senior center staff who neither had a background in lifestyle intervention nor were healthcare professionals.

In the Latino Education Project, lay health educators from Hispanic communities were trained to organize local media campaigns and to deliver group sessions for older adults, but they were also involved in the whole intervention development [23]. In the People Exercising Program, the training of laypersons took part in daylong interactive face-to-face workshops and was delivered by project investigators [31].

#### 3.1.4. Strengthening Competence and Awareness in the Target Population

All fourteen studies used strategies to strengthen the competence and awareness in the target population. This category included all activities that directly addressed the individuals in the target population. Accordingly, the format and intensity of these strategies varied across the studies from single information events to regular classes. Local media campaigns were often initiated by the community coalitions, with laypersons supporting the distribution of the program materials. For example, in the Groningen Lifestyle Intervention for Seniors [33], older adults from a socioeconomically disadvantaged community were involved in the development of the intervention and later functioned as role models for posters, radio spots, and interviews. They were also engaged in the distribution of handbooks and flyers. In the Latino Education Project, lay health educators were trained to organize local media campaigns [23]. Some of the projects organized single events (e.g., public lectures) or awareness weeks to capture the attention of the target group [25,26,30]. In the projects described by Peterson et al. [37] and Jitramontree et al. [27], community surveys were conducted and the results were locally disseminated to raise awareness in the population.

Several projects included behavior-oriented intervention components, mostly organized as regular group sessions. As already mentioned, some of these used existing evidence-based programs [28,31,37,38], or the program was developed according to national guidelines [32]. The Escalante Health Partnerships also included screenings for hypertension, diabetes, and high cholesterol.

#### 3.1.5. Allocation of Financial Resources

Four projects included the allocation of financial resources as a strategy for capacity building. In the project described by Abuzahra and Hinterberger, a fund for health-related projects was established [25] and project municipalities were encouraged to apply for funding. Local projects had to show an innovative approach in addressing the health of the local older population. However, there was no information about the amount of money allocated. Another project included a fund for the 13 municipalities involved [35]. The fund volume amounted to €45,000 and was provided by the general funding organization and the participating municipalities. In the AACTF project, local lead agencies were provided with $16,000 over a two-year period to support their efforts [32]. Similarly, local agencies received $19,000 over five years for the implementation of the Stepping On program [36,37].

### 3.2. Success of the Strategies

As many of the included studies were case studies, only three analyzed the efficacy of the interventions. West et al. [38] analyzed the efficacy of the Diabetes Prevention Program implemented by lay health educators. The results of this cluster randomized trial showed greater weight loss in the intervention groups at the end of the 12-week program compared to an untreated control group. The other cluster randomized trial included in the review analyzed whether an enhanced prevention support system (i.e., more intensive capacity building) would improve the effects of a fall prevention program compared to a standard support system and an untreated control group [36,37]. The results showed a significant reduction in the fall injury incidence in both intervention groups, but no beneficial effects of the enhanced support group over the standard support group. Thus, intensified capacity building did not improve the program effects. Although two cohort studies reported a high reach in the target population, no or inconsistent effects on PA and fruit and vegetable consumption were found [33,34]. The study by Layne et al. [19] analyzed the implementation differences of the People Exercising Program. It compared the use of professional versus lay health educators and showed that both implementation strategies were feasible.

None of the included publications assessed community capacities as an explicit endpoint or outcome. Implementation success was, however, evaluated in some studies. For example, the AACTF study conducted a survey to assess a coalition´s stage of development, as well as coalition leaders’ competence, performance, support, and control [32]. Several studies summarized their success in the form of lessons learned in terms of facilitating factors and barriers in the capacity building process with regard to community coalitions and networks (Table 3).

In summary, facilitating factors focused on the structure of community coalitions and networks (e.g., early involvement of cooperation partners, written agreements), the involvement of diverse local partners and the synergies of existing networks. Barriers dealt with more negative aspects of existing networks (e.g., interlocking of partners, competition between partners, and unclear allocation of roles).

## 4. Discussion

The aim of this literature review was to identify capacity building strategies for the promotion of PA among older adults and to find evidence for the success of the strategies. The 14 studies included provided many practical examples that may guide future activities in this field. We classified the strategies used into five main groups: (1) Community coalitions and networks; (2) training of professionals; (3) training of laypersons; (4) strengthening the competence and awareness in the target population; and (5) allocation of financial resources. All studies combined strategies from at least two of these groups. Strategies to build community coalitions and strengthen the competence and awareness in the target population were applied in almost all of the identified studies. Nevertheless, although studies used the same groups of strategies, their overall aim of capacity building differed. Some used capacity building to facilitate the implementation and dissemination of an existing program, while others aimed to strengthen community capacities for PA in general. The different approaches described in the studies seemed to be feasible and acceptable, with a large reach among the target population and high participant satisfaction ratings being reported. Several accounts of lessons learned were provided describing factors attributed to implementation success. The early involvement of community partners and the formation of broad inter-sectoral alliances with clear roles and responsibilities, as well as a transparent flow of information, were some of the suggestions given in this respect. However, only weak evidence concerning the intervention outcomes was observed. Many studies did not evaluate intervention effects in terms of an increase of PA or other relevant health outcomes. Among the studies that assessed the intervention effects, the results were inconsistent. The only study that systematically compared different levels of capacity building did not find any beneficial effect of an enhanced support system over a standard support system [36].

Some limitations nonetheless have to be considered while interpreting the results. Community capacity building is often carried out invisibly due to its relative unpopularity with funding authorities [39]. Furthermore, capacity building is sometimes subsumed under different concepts, e.g., community empowerment or community development [14]. We addressed this issue by including known community capacity strategies (e.g., building community coalitions) and other concepts closely related to community capacity building based on the WHO definition in the search strategy. However, it is possible that we missed some studies which used community capacity building strategies indirectly, without explicit reference, operationalization as the study aim or method used. Further, the literature review also included grey literature. While such publications often lack methodological quality and are not peer-reviewed, their inclusion has been shown to be effective to control for publication bias, as well as overestimating effects [40]. Nevertheless, publication bias cannot be ruled out in this review since project reports may lean towards reporting positive results to funding authorities, possibly leaving out negative aspects. The quality assessment showed that the studies included in the review were of low to moderate quality, and that at times important information such as sample size was lacking. While this inclusive search strategy served well for the purpose of the study, more rigorous research is needed in order to be able to evaluate the effectiveness of community capacity building approaches in the context of promoting PA in the older population.

While our review focused on capacity building in a specific field of research (PA promotion among older adults), our understanding of capacity building strategies might benefit from findings from other fields beyond our focus. For example, Marlier et al. [41] compared communities enhancing their capacity through community coalitions (intervention group) with control communities. Ten years after implementing community coalitions, higher sports engagement and more time spent on PA were reported in the intervention group. Furthermore, Anderson et al. [42] conducted a systematic review to assess the effects of community coalition-driven interventions in improving health status and health behaviors. Overall, the authors found that educating lay community health workers and initiating professionally led group-based health education interventions have beneficial effects on health status outcomes, as well as health behaviors. However, due to the lack of detailed information on the coalitions included in their systematic review, Anderson et al. concluded that it was not possible to make a definite judgement on whether community coalitions add extra value to community health interventions. Similarly, O’Mara-Eves et al. [43] reported positive effects on health and health behaviors of interventions aiming to increase community engagement and participation after conducting a meta-analysis of respective studies on interventions for disadvantaged groups. However, the data were not sufficient to determine whether certain strategies were more successful than others.

## 5. Conclusions

Although community capacity building is recommended as a core strategy for sustainable and equitable health promotion, little guidance on how to enhance community capacities exists. This literature review identified five main groups of community capacity building approaches with respect to PA promotion among the elderly. Building community coalitions and strategies to strengthen the competence and awareness in the target population were the most prominent approaches reported in the literature identified. Since the overall quality of the studies was only moderate, more rigorous research is needed to enable more comprehensive evaluations of the effectiveness of community capacity building approaches in the context of health promotion, specifically for promoting PA in the older population.

## Figures and Tables

**Table 1:** Characteristics of the included studies.

Authors	Study Design	Setting	Target Group Sample Size	Quality Appraisal (Number of ‘Yes’)
Sotomayor et al. 2007 [23]	Case study	Nueces County, South Texas, USA	Middle-aged and elderly Latinos N = not specified	5/10
Kolb et al. 2011 [24]	Case study	Area of Vienna, Austria	Inactive, older adults N = 12 (focus group)	7/10
Abuzahra & Hinterberger 2012 [25]	Case study	5 communities in Steiermark, Austria	Older adults (≥50 years) N = not specified	4/10
Sundermeier 2011 [26]	Case study	8 communities in the Rhein-Sieg area, Germany	Older adults (≥60 years)/ N = not specified	6/10
Jitramontree et al. 2015 [27]	Case study	Community in West Bangkok, Thailand	Older adults (≥60years), family members, PHNs, health volunteers, community leader N = 80	5/10
Zgibor et al. 2016 [28]	Survey	54 communities in Allegheny County, PA, USA	Older adults (≥50 years) N = 462	6/10
Nunez et al. 2003 [29]	Survey	Escalante, UT, USA	Older adults (≥50 years) N = 135	4/10
FGG 2012 [30]	Survey	Urban district Dortmund-Eving, Germany	Older adults, low SES, migrant background, or limited mobility N = 55	4/10
Layne et al. 2008 [31]	Survey	Senior centers, community centers, and churches in New England, USA	Older adults (≥50 years) N = 244 trainers N = 2217 participants	3/10
Hooker & Cirill 2006 [32]	Cohort study	28 counties in California, USA	Older adults (≥50 years) N = 167 (PA class participants) N = 90 (coalition members)	4/10
Luten et al. 2016 [33]	Cohort study (CBA)	Disadvantaged community in Groningen, The Netherlands	Older adults (≥50 years) N = 641	8/10
Neuhold 2008, Reis-Kling-spiegl 2008 [34,35]	Cohort study (BA)	13 communities in Graz and Voitsberg, Austria	Older adults (≥60 years)/ N = 908	5/10
Guse et al. 2015/ Peterson et al. 2015 [36,37]	CRCT	20 Counties in Wisconsin, USA	Older adults (≥65 years)/ N = 817	6/11
West et al. 2011 [38]	CRCT	15 senior centers in Arkansas, USA	Older adults (≥60 years), obese (BMI ≥ 30) N = 228	6/11

PA physical activity; PHN public health nurses; SES socioeconomic status; CBA controlled before-and-after study; BA before-and-after study; CRCT cluster randomized controlled trial; BMI body mass index.

**Table 2:** Intervention components, outcome measures, and results of the included studies.

Authors	Intervention Components	Outcome Measures	Results
*Case studies*			
Sotomayor et al. 2007 [23]	*Community coalitions and networks*: Community-wide health forums and coalitions consisting of elected officials, older adults, representatives of community groups and agencies *Training of laypersons*: Training of lay health educators *Strengthening competence and awareness in the population*: Local media campaigns; Group sessions for residents providing health-related knowledge and social support	None	Anecdotal evidence that the activities improved the health of the target group by encouraging use of appropriate health services
Kolb et al. 2011 [24]	*Community coalitions and networks*: Building inter-sectoral alliance for PA; Quality standards for PA programs for older adults *Strengthening competence and awareness in the population*: Launch of a website for PA for older adults	- Barriers and factors of success - Recommendations for transferability	- Strengthening of inter-sectoral capacities successful - Model for participatory planning process - Implementation of initial actions - Handing over the responsibility to regional partners to achieve sustainability
Abuzahra & Hinterberger 2012 [25]	*Community coalitions and networks*: Community group formation in five municipalities including representatives of regional development and tourism agencies *Strengthening competence and awareness in the population*: Lectures and classes on PA, nutrition, sexuality and vitality *Allocation of financial resources*: Local projects applied for funding	- Barriers and factors of success	- Strengthening competence of target group successful - Building of community-based coalitions/networks successful - Independence and sustainability of networks secured - Transfer not reached
Sundermeier 2011 [26]	*Community coalitions and networks*: Community-based working groups were formed during meetings with local key persons in four pilot municipalities *Training of professionals*: Training of management skills and public relations issues for network members; Training of PA class instructors *Strengthening competence and awareness in the population*: Information events and special days for mapping existing exercise/PA programs	- Assessment of sustainability - Lessons learned	- Installed networks remain beyond project duration - Communities, operators and target group sensitized
Jitramontree et al. 2015 [27]	*Community coalitions and networks*: Community participatory planning process with community leader, public health nurses, public health volunteers, older adults, family members *Strengthening competence and awareness in the population*: Risk assessment among older adults; Dissemination of results via community broadcast system; Development and dissemination of a fall prevention handbook; Exercise and cane use training program; Home visits; Reminder calls	Perceived benefits of the program	- Age-friendly handbook improved communication about falls - Mutual learning facilitated by group sessions - Motivation from telephone reminders
*Cross-sectional surveys*			
Zgibor et al. 2016 [28]	*Community coalitions and networks*: Formation of research-funding agency partnership; Formalized site selection process *Training of professionals*: Training for instructors *Strengthening competence and awareness in the population*: PA and health education group sessions	- Participant satisfaction - Lessons learned	- High participant satisfaction - Partnering with organizations having an existing infrastructure supports program delivery at the community level
Nunez et al. 2003 [29]	*Community coalitions and networks*: University-community partnership (university college of nursing, local health department, and a community action agency) *Strengthening competence and awareness in the population*: PA courses for older adults; Health education (e.g., healthy nutrition); Screening for hypertension, diabetes and high cholesterol	- Health-related quality of life (SF-36)	- Higher SF-36 scores compared to national norms
FGG 2012 [30]	*Community coalitions and networks*: University-led community coalition including the local senior office, (intercultural) community centers, peers and other stakeholders *Strengthening competence and awareness in the population*: Public lectures on health promotion; Provision of PA and healthy eating classes	- Health status and behavior (SF-12) - Barriers and factors of success	- Participants showed awareness of health promoting behavior and mostly good health - Involvement of laypersons and members of the target group as facilitating factor - Word of mouth important for gaining access to the target group - Establishing PA among the older adults as a regular topic in relevant working groups
Layne et al. 2008 [31]	*Training of professionals*: Train-the-trainer workshops for PA class instructors (‘leadership training’) *Training of laypersons*: Same training as for the professionals *Strengthening competence and awareness in the population*: PA classes for older adults	- Feasibility: 75% of the trainers providing at least two classes within 1 year - Dissemination: number of classes provided at the end of a 2-year period	- Feasibility proven: N = 244 completed the instructor training workshop, 79% of the trainers at least two classes within 1 year - No implementation differences between professional and layperson trainers - Dissemination: 97 classes provided after 2 years
*Cohort studies*			
Hooker & Cirill 2006 [32]	*Community coalitions and networks*: Coalition led by administrative and program personnel from local health departments and area agency on aging *Training of professionals*: Training of PA class instructors *Strengthening competence and awareness in the population*: PA classes for older adults *Allocation of financial resources*: Funding for local implementation	- Coalition self-assessment survey - Number of new exercise classes - Functional fitness assessment	- High ratings for coalition functioning - 153 new exercise classes provided - Improvements in low back/hip range of motion, agility/dynamic balance, leg strength, and upper arm strength among class participants - No adverse events
Luten et al. 2016 [33]	*Community coalitions and networks*: Local healthcare professionals and peers were involved in the intervention development and implementation *Strengthening competence and awareness in the population*: Local media campaign (posters, radio spots, radio interviews, advertorials and press reports, newsletters, flyers, Goud Leven guide, website)	- Reach - Change in self-reported PA/fruit and vegetable consumption after 3 and 9 months	- Large proportion of the participants were reached - No effects on total PA and fruit and vegetable consumption compared to control group
Neuhold 2008/Reis-Klingspiegl 2008 [34,35]	*Community coalitions and networks*: Senior networks and platforms in 13 municipalities *Training for professionals*: Management skills training for project leaders *Allocation of financial resources*: Local projects applied for funding	- Social mobilization and activation of target group - Change of norms, values and attitudes - Individual health potential and health-related quality of life	- Social mobilization and activation of target group succeeded - No effect on norms, values and attitudes - Slight increase in physical fitness and life satisfaction - Health-related quality of life stable on high level
*Cluster randomized controlled*			
Guse et al. 2015/Peterson et al. 2015 [36,37]	*Community coalitions and networks*: Research-led coalition with aging units and local health offices; Provision of technical assistance *Training of professionals*: Fall prevention instructor training, group facilitation skills, marketing and recruitment techniques *Strengthening competence and awareness in the population*: Local events (Fall Prevention Awareness Days); Dissemination of local survey results; Fall prevention classes for older adults *Allocation of financial resources*: Funding for local implementation	- Fall injuries incidence	- Significant reduction in fall injury incidence in in the standard and enhanced support communities (9% and 8% respectively) compared to control communities - No difference between standard and enhanced support communities
West et al. 2011 [38]	*Training of laypersons*: Training of lay health educators *Strengthening competence and awareness in the population*: 12-week group-based standardized lifestyle intervention	- Weight loss - Treatment adherence - Participant satisfaction	- Significant weight loss (intervention group = −3.7 kg vs. control group = −0.3 kg) - High attendance (mean no. of attended sessions: 9.1 of 12) - High satisfaction with program among participants

PA—physical activity.

**Table 3:** Facilitating factors and barriers in the capacity building process.

Facilitating Factors	Barriers
Early involvement of a constant local cooperation partner that takes over responsibility	Reliance (e.g., financial) on a single project partner
Written agreements with project partners to improve engagement and collaboration	Administrative and political interlocking between network partners
Conscientious choice of project partners and stakeholders (e.g., build a group that is able to take decisions, inclusion of stakeholders from all community sectors)	Conflicts of interest inside the network
Strong integration of local politics	Competitive thinking among the network partners
Target group involvement during planning phase	Change of persons in charge within the project team
Using synergies from existing networks	A narrow project schedule
Allocation of financial resources by the municipality	Difficulties in understanding the workshop content among laypersons
Transparent information flow to keep the partners informed	Unclear allocation of roles by local authorities and other organizations involved

## Supplementary Materials

The following are available online at www.mdpi.com/1660-4601/14/9/1058/s1, Table S1: Search strategy (Web of Science), Table S2: Search strategy (PsycInfo), Table S3: Quality assessment of case studies, Table S4: Quality assessment of randomized controlled studies, Table S5: Quality assessment cross-sectional studies, Table S6: Quality assessment cohort studies, Table S7: Overview of applied quality assessment instruments.
