# Supplementary material for: Community Capacity Building for Physical Activity Promotion among Older Adults—A Literature Review
Source: Int J Environ Res Public Health. 2017 Sep 13;14(9):1058. doi: 10.3390/ijerph14091058 (PMC5615595; doi:10.3390/ijerph14091058)
Supplement: Supplementary file 1 [file ijerph-14-01058-s001.pdf]

Table S1. Search strategy (Web of Science)

#1	TS=("capacity building" OR "community development" OR "community empowerment" OR "community network*" OR "coalition building" OR "community capacit*" OR "community building" OR "community participation" OR "community partnership*" OR "community leadership" OR "community coalition") DocType=All document types; Language=All languages;
#2	TS=(increase OR promote OR enhance OR improve OR develop OR up OR build) DocType=All document types; Language=All languages;
#3	TS=("community readiness") DocType=All document types; Language=All languages;
#4	#2 AND #3 DocType=All document types; Language=All languages;
#5	#1 OR #4 DocType=All document types; Language=All languages;
#6	TS=(elderly OR "older adults" OR senior OR "older people" OR ageing) DocType=All document types; Language=All languages;
#7	TS=(communit* OR municipalit* OR environment* OR neighborhood OR "third sector") DocType=All document types; Language=All languages;
#8	TS=("health promotion" OR prevention) DocType=All document types; Language=All languages;
#9	#5 AND #6 AND #7 AND #8 DocType=All document types; Language=All languages;

Date: 03.03.2017, Languages: English, German

Table S2. Search strategy (PsycInfo)

1	("capacity building" or "community development" or "community empowerment" or "community network*" or "coalition building" or "community capacity*" or "community building" or "community participation" or "community partnership*" or "community leadership" or "community coalition").af.
2	limit 1 to ((english or german) and yr="1997 - 2017")
3	(increase or promote or enhance or improve or develop or up or build).af.
4	limit 3 to ((english or german) and yr="1997 - 2017")
5	"community readiness".af.
6	limit 5 to ((english or german) and yr="1997 - 2017")
7	4 and 6
8	2 or 7
9	(elderly or "older adults" or senior or "older people" or ageing).af.
10	limit 9 to ((english or german) and yr="1997 - 2017")
11	(communit* or municiplait* or environment or neighborhood or "third sector").af.
12	limit 11 to ((english or german) and yr="1997 - 2017")
13	("health promotion" or prevention).af.
14	limit 13 to ((english or german) and yr="1997 - 2017")
15	8 and 10 and 12 and 14

Date: 03.03.2017, Languages: English, German

Table S3. Quality assessment of case studies (Critical Appraisal of a case study (CEBMa))

Syudy	1	2	3	4	5	6	7	8	9	10	**Results**
**Sotomayor et al. 2007**	Yes	NA	Yes	Ct	NA	NA	NA	Yes	Yes	Yes	5/10
**Kolb et al. 2011**	Yes	Yes	Yes	Ct	Yes	Ct	No	Yes	Yes	Yes	7/10
**Abuzahra & Hinterberger 2012**	Yes	NA	Yes	Ct	No	Ct	Ct	Yes	Ct	Yes	4/10
**Sundermeier 2011**	Yes	Yes	Yes	Ct	No	Ct	Ct	Yes	Yes	Yes	6/10
**Jitramontree et al. 2015**	No	Yes	Yes	Ct	Yes	Yes	Ct	Yes	No	No	5/10

Ct can’t tell; NA not applicable

*Critical appraisal of a case study, appraisal questions

1. Did the study address a clearly focused question / issue?

2. Is the research method (study design) appropriate for answering the research question?

3. Are both the setting and the subjects representative with regard to the population to which the findings will be referred?

4. Is the researcher´s perspective clearly described and taken into account?

5. Are the methods for collecting data clearly described?

6. Are the methods for analyzing the date likely to be valid and reliable? Are quality control measures used?

7. Was the analysis repeated by more than one researcher to ensure reliability?

8. Are the results credible, and if so, are they relevant for practice?

9. Are the conclusions drawn justified by the results?

10. Are the findings of the study transferable to other settings?

Table S4. Quality assessment of randomized controlled studies (Section H Critical Appraisal Form (Stanford School of Medicine))

Study	1	2	3	4	5	6	7	8	9	10	11	Results
**Smith West et al. 2011**	Yes	Yes	Yes	NR	No	Yes	NR	Yes	No	Yes	NR	6/11
**Guse et al. 2015;**	Yes	Yes	Yes	NR	No	Yes	NA	Yes	No	Yes	No	6/11
**Peterson et al. 2016**												

NA not applicable; NR not reported

*Intervention trials, Section H: Specific methodological issues

1. Randomization properly done
2. Baseline comparability reported
3. Same data collection for all arms
4. Subjects blinded to treatment assignment
5. Care givers blinded to treatment assignment
6. Treatments clearly described
7. Co interventions monitored
8. Compliance monitored and equal in all groups
9. Side effects assessed
10. Outcomes defined, measureable, and valid
11. Blind assessment of outcomes

Table S5. Quality assessment cross-sectional studies (Section Q Critical Appraisal Form (Stanford School of Medicine))

Study	1*	2	3	4	5	6	7	8	9	10	Results
**Nunez et al. 2003**	NR	Yes	Yes	NR	Yes	No	No	No	Yes	NA	4/10
**FFG 2011**	No	Yes	NR	Yes	Yes	No	No	No	Yes	No	4/10
**Layne et al. 2008**	No	NA	Yes	NA	Yes	NA	NA	NR	Yes	NA	3/10
**Zgibor et al. 2016**	Yes	Yes	Yes	Yes	Yes	NR	NR	No	Yes	No	6/10

NA not applicable; NR not reported

*Cross-sectional study, Section Q: Specific methodological issues

1. Similar sampling procedures for all subjects
2. Similar ascertainment of exposure for all subjects
3. Similar referral and diagnostic procedures for all subjects
4. Diagnostic criteria for diseased clear, precise, and valid
5. Characteristics of subjects at enrollment reported
6. All aspects of exposure measured (level dose, duration)
7. Co-exposure measured
8. Recall bias controlled
9. Data collection valid and reliable
10. Effect of duration of disease discussed

Table S6. Quality assessment cohort studies (Section K Critical Appraisal Form (Stanford School of Medicine))

Study	1*	2	3	4	5	6	7	8	9	10	Results
**Hooker & Cirill 2006**	Yes	No	Yes	NR	NR	No	No	Yes	Yes	No	4/10
**Neuhold 2008**	Yes	Yes	No	Yes	NA	NR	No	Yes	Yes	No	5/10
**Luten et al. 2016**	Yes	Yes	Yes	Yes	Yes	Yes	No	Yes	Yes	No	8/10

NA not applicable; NR not reported

*Cohort or prognostic study, Section K: Specific methodological issues

1. Zero time identified
2. Baseline comparability reported
3. Same data collection in all groups
4. Important baseline variables measured, valid and reliable
5. Exposure adequately measured (previous, at entry, during study)
6. Regular visits during follow up
7. Co exposure monitored
8. Duration of follow-up adequate
9. Outcomes defined, measureable, and valid
10. Blind assessment of outcomes

Table S7. Overview of applied quality assessment instruments

**Assessing scientific admissibility and merit of published articles. Critical appraisal form (Stanford School of Medicine)**	
**Section B: General methodological issues**	**Section H: Specific methodological issues - randomized controlled studies**
1. A priori hypothesis clearly stated	1. Randomization properly done
2. Source population identified	2. Baseline comparability reported
3. Inclusion criteria described and appropriate (same in all groups)	3. Same data collection for all arms
4. Exclusion criteria described and appropriate (same in all groups)	4. Subjects blinded to treatment assignment
5.Number of excluded or refusal (before study) reported	5. Care givers blinded to treatment assignment
6. Withdrawals (during study) reported, explained, and reasonable	6. Treatments clearly described
7. Withdrawals equal in groups	7. Co interventions monitored
8. Sample size preplanned to provide adequate statistical power	8. Compliance monitored and equal in all groups
9. Statistical analysis appropriate	9. Side effects assessed
10. Adjustment for multiple comparisons	10. Outcomes defined, measureable, and valid
11. Adjustment for important variables	11. Blind assessment of outcomes
12. Results verifiable from raw data	
**Section K: Specific methodological issues - cohort studies**	**Section Q: Specific methodological issues - cross-sectional studies**
1. Zero time identified	1. Similar sampling procedures for all subjects
2. Baseline comparability reported	2. Similar ascertainment of exposure for all subjects
3. Same data collection in all groups	3. Similar referral and diagnostic procedures for all subjects
4. Important baseline variables measured, valid and reliable	4. Diagnostic criteria for diseased clear, precise, and valid
5. Exposure adequately measured (previous, at entry, during study)	5. Characteristics of subjects at enrollment reported
6. Regular visits during follow up	6. All aspects of exposure measured (level dose, duration)
7. Co exposure monitored	7. Co-exposure measured
8. Duration of follow-up adequate	8. Recall bias controlled
9. Outcomes defined, measureable, and valid	9. Data collection valid and reliable
10. Blind assessment of outcomes	10. Effect of duration of disease discussed
**Critical appraisal of a case study (Center for Evidence-Based Management)**	
1. Did the study address a clearly focused question / issue?	
2. Is the research method (study design) appropriate for answering the research question?	
3. Are both the setting and the subjects representative with regard to the population to which the findings will be referred?	
4. Is the researcher´s perspective clearly described and taken into account?	
5. Are the methods for collecting data clearly described?	

6. Are the methods for analyzing the date likely to be valid and reliable? Are quality control measures used?
7. Was the analysis repeated by more than one researcher to ensure reliability?
8. Are the results credible, and if so, are they relevant for practice?
9. Are the conclusions drawn justified by the results?
10. Are the findings of the study transferable to other settings?
